# The Correlations between Microstructures and Color Properties of Nanocrystalline Cellulose: A Concise Review

**DOI:** 10.3390/polym16192774

**Published:** 2024-09-30

**Authors:** Keming Zhu, Xing Zhou, Dong Wang, Dexiang Li, Wanqing Lei, Changqing Fang, Jingbo Hu, Rubai Luo

**Affiliations:** 1 School of Mechanical and Precision Instrument Engineering, Xi’an University of Technology, Xi’an 710048, China; kemingzhu246@163.com; 2 Faculty of Printing, Packaging Engineering and Digital Media Technology, Xi’an University of Technology, Xi’an 710048, China

**Keywords:** cellulose nanocrystals, liquid crystal, color, chirality, self-assembly

## Abstract

Cellulose nanocrystals (CNCs) are a green resource which can produce photonic crystal films with structural colors in evaporation-induced self-assembly; CNC photonic crystal films present unique structural colors that cannot be matched by other colored materials. Recently, the mechanisms of CNC photonic crystal films with a unique liquid crystal structure were investigated to obtain homogenous, stable, and even flexible films at a large scale. To clarify the mechanism of colorful CNC photonic crystal films, we briefly summarize the recent advances from the correlations among the preparation methods, microstructures, and color properties. We first discuss the preparation process of CNCs, aiming to realize the green application of resources. Then, the behavior of CNCs in the formation of liquid crystal phases is studied, considering the influence of the CNCs’ size and shape, surface properties, and the types and concentrations of solvents. Finally, the film formation process of CNCs and the control of structural colors during the film formation are summarized, as well as the mechanisms of CNC photonic crystal films with full color. In summary, considering the above factors, obtaining reliable commercial CNC photonic crystal films requires a comprehensive consideration of the subsequent preparation processes starting from the preparation of CNCs.

## 1. Introduction

Throughout the evolutionary history of organisms, plants and animals have developed sophisticated structures and diverse pigmentation cells to generate colors that function for camouflage, as warnings, and to communicate information [1]. Colors derived from these structures are especially notable in insects, birds, plants, and other organisms, with their hues resulting from the materials’ internal architecture causing light scattering, interference, and other physical phenomena, rather than from pigments. Consequently, such structurally produced colors do not undergo the biological degradation and fading characteristic of chemical pigments [2]. The concept of photonic crystals was independently proposed in 1987 by the research teams of John and Yablonovitch [3,4]. Advancements in nanotechnology, micro- and nanofabrication techniques, and microstructure observation techniques have significantly bolstered both theoretical and applied research into photonic crystals in recent years.

The photonic crystal structure can be divided into three types, 1D, 2D, and 3D, according to the different dimensions of its periodic arrangement. Among the preparation of these three types of photonic crystals, the technology that is currently more mature and widely applied is the biomimetic fabrication of photonic crystals [5]. The manufacturing of photonic crystals draws inspiration from various natural structures, such as the iridescent wings of the Morpho butterfly, the vibrant feathers of peacocks, and the intricate armor of beetles [6,7]. Notably, the carapace of the golden-like Chrysina aurigans scarab beetles exhibits a remarkable spiral structure [6]. This structure arises from the orderly parallel arrangement of chitin nanofibers within the same layer of the beetle’s exoskeleton, with adjacent layers rotated clockwise by a specific angle. These animal-inspired one-dimensional photonic crystals have become a research focus due to their simple fabrication and low cost [8,9].

Natural materials that serve as the basic units for biomimetic photonic crystals have drawn a large amount of attention recently. It was found that cellulose nanocrystals (CNCs) are one-dimensional chiral nanorods with nanoscale dimensions that can spontaneously exhibit liquid crystal behavior at certain concentrations [10,11]. CNCs can be spontaneously organized into left-handed helically nematic liquid crystals with a structure similar to that of beetles’ Bouligand structures [12]. After drying, CNC suspensions can form solid films with a left-handed helical structure, displaying the optical properties of chiral nematic liquid crystals [13,14]. This unique optical property is attributed to the periodic spiral arrangement of CNCs, which constitutes a one-dimensional photonic crystal [15]. The structural color variation in one-dimensional photonic crystals, as per the Bragg equation, is primarily influenced by the distance between the cellulose layers [16]. CNCs, due to variations in their preparation methods, often exhibit differences in shape, size, surface charge density, and so on. These differences are crucial in influencing the distance between the cellulose layers during the self-assembly process [17,18,19]. Although the mechanism of CNC structural color has been discovered, providing theoretical support for the preparation of CNC-based photonic crystals, the current preparation methods of CNC-based photonic crystal materials mainly rely on microfluidic techniques or evaporation-induced self-assembly, which are difficult to scale up. Moreover, CNC photonic crystals prepared by these methods are prone to defects, necessitating the development of new preparation methods to overcome these defects and expand the functionality of the materials. Additionally, there is a lack of in-depth understanding of the mechanism of CNC chiral nematic phase formation, which requires further research into the influence of different factors (such as size, shape, surface charge, etc.) on the chiral nematic phase structure and optical properties.

This review primarily aims to discuss the preparation of CNCs, the principle of liquid crystal phase formation, and the control of self-assembly. Firstly, the preparation of CNCs is studied, and different preparation methods affect the shape, surface charge, etc., of CNCs, laying the foundation for subsequent commercial applications. Subsequently, the important roles of the CNCs’ shape, size, and surface charge in the formation of the liquid crystal phase are discussed, based on which CNC self-assembly and the regulation of optical properties will be better understood. Additionally, the rapid self-assembly of CNCs is summarized, which is needed for future applications. The focus is on the correlations between the microstructures and color properties of nanocrystalline cellulose. It is found that by compositing with other materials or performing post-processing, the mechanical strength and toughness of CNC-based photonic crystal materials can be enhanced while also achieving the control of the CNCs’ pitch. In the future, it is necessary to deepen the research on CNC mechanisms, develop new self-assembly methods, and promote the development and application of materials.

## 2. Preparation of Cellulose Nanocrystals

**Figure 1:**
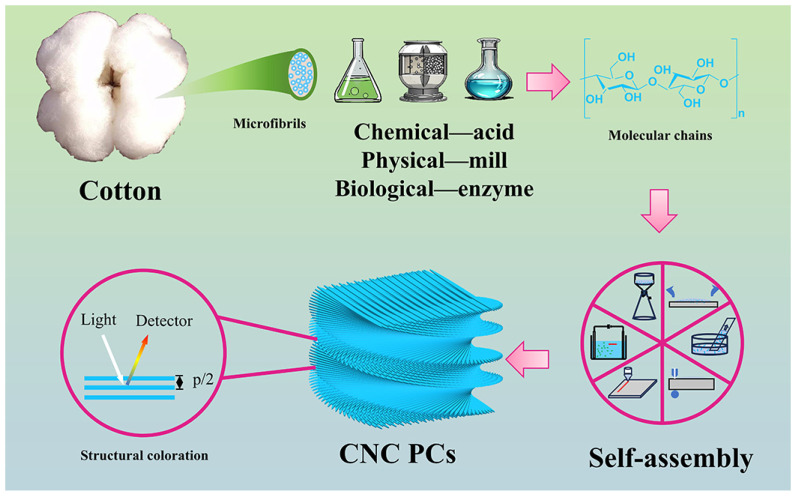
Scheme of the preparation of CNC PCs with functional performance from natural materials.

As shown in Figure 1, CNCs are nanoscale crystals extracted from natural cellulose fibers (including cotton, wood pulp, etc.), possessing characteristics such as a high specific surface area, high strength, and high rigidity. The methodologies for cellulose nanocrystal preparation can be categorized into chemical, physical, and biological approaches, encompassing techniques such as acid hydrolysis, ionic liquid treatment, oxidation, ball milling, and enzymatic hydrolysis [20,21,22,23]. CNCs can be self-assembled into periodic cyclical structures (PCs) to obtain various functional CNC films and their composites. Table 1 summarizes the principles, product characteristics, and advantages and disadvantages of the preparation methods.

**Table 1:** Preparation methods, reaction conditions, product characteristics, and yields of CNCs.

Method	Raw Materials	Reaction Condition		CNCs Characteristics			Yield/%	Ref.
		Time	TEMP/°C	L/nm	W/nm	Zeta/mV		
Inorganic acid hydrolysis	MCC/H_2_SO_4_	60 min	45	141.3	6.5	−49.4	32.7	[24]
	MCC/H_2_SO_4_	10 min	70	200–300	5–10	NA	25	[25]
	BEKP/FA/FeCl_3_	6 h	95	50–200	5–20	−6.02	75.7	[26]
Organic acid hydrolysis	MCC/ZnCl_2_/CA	8 h	25	40–75	6–10	−18.9	61.4	[27]
	ECF/NaOH/OA	8 h	90	310	16.5	−34.2	59	[28]
Solid acid hydrolysis	MCC/OA	4 h	80	350	10	NA	59	[29]
	BEP/FeCl_3_·6H_2_O	10 min	70	322	10–27	−14.1	80.1	[30]
Ionic liquid treatment method	CC/[Bmim][HSO_4_]	1.5 h	100	166	NA	−36.1	40.1	[31]
	MCC/[DEAPA][Hex]	3 h	80	410	38	−4.2	24	[32]
	MCC/DMSO/ [Bmim][HSO_4_]	1.5 h	90	757	50	−20.0	60	[33]
Oxidation method	SBKP/TEMPO/NaBr/NaClO	10 h	25	185	3.5	−54.0	94	[34]
	Hardwood	4.5 h	170	150–250	5	−43.0	87	[35]
	MCC/H5IO_6_/KOH	14 days	25	116–120	3.7–5.3	−25.5	40.1	[36]
	MCC/NaIO4/FeSO_4_·7H_2_O	10 h	45–60	275	22.3	−27.2	51.2	[37]
Wet ball milling	MCC	0.5–16 h	25	120–400	3–10	NA	20	[38]
	MCC/H_3_PO_4_	15–60 min	25	230–290	8–10	−23.0	76	[39]
Enzymolysis approach	MCC	72 h	50	347–350	35.5	NA	13.1	[40]
	BEPF	21 h	50	600	30	NA	NA	[21]

Among the various methods, sulfuric acid hydrolysis stands out as one of the earliest and most frequently employed techniques, utilizing sulfuric acid to cleave the glycosidic bonds and disrupt the amorphous regions of cellulose, thereby yielding a more stable CNC suspension [24]. Following hydrolysis, methods such as freeze drying and supercritical drying are employed to remove water without damaging the nanocrystalline structure, resulting in dried CNC products. Besides sulfuric acid, other acids such as hydrochloric acid, phosphoric acid, and mixed acid hydrolysis have also been explored. However, these methods are not without drawbacks, including issues such as equipment corrosion, the requirement for large volumes of water, and environmental pollution.

To overcome the drawbacks of acid hydrolysis, researchers improved the hydrolysis method. The one-pot synthesis of CNCs offers significant environmental benefits. This method eliminates the need for hazardous chemicals such as strong alkalis or organic solvents, thereby reducing environmental pollution. Additionally, the elimination of high-temperature and -pressure treatments lowers energy consumption. Furthermore, the one-pot synthesis of CNCs simplifies the preparation steps, facilitating the recycling of wastewater [25]. In addition, weak acids such as formic acid can also optimize the drawbacks of traditional strong acid hydrolysis methods. Du et al. [26] enhanced formic acid hydrolysis by catalyzing it with FeCl_3_, achieving a hydrolysis efficiency of 75% and producing CNCs with high crystallinity and thermal stability. This method of adding FeCl_3_ not only enhances the hydrolysis efficiency of formic acid but also mitigates some HCl environmental pollution issues.

More and more environmentally friendly materials have been selected for the preparation of CNCs. Aqueous solutions of ZnCl_2_ and citric acid (CA) can hydrolyze at room temperature to produce carboxylated CNCs. Both ZnCl_2_ and CA are environmentally friendly raw materials, and the process does not require high temperatures and pressures, thereby avoiding the acid and organic solvent contamination of traditional methods [27].

Chenrian et al. [28] employed a chemical–mechanical strategy that involved pretreating the cellulose with NaOH and NaClO_2_ to remove noncellulosic components, followed by mild oxalic acid hydrolysis using steam explosion and homogenization, resulting in CNCs with high crystallinity and thermal stability. Organic acid hydrolysis is less corrosive, and organic acids can be readily recovered through recrystallization. However, this method is hindered by its low reaction efficiency and the need for feedstock pretreatment. Solid acid hydrolysis is a novel approach for CNC preparation. Song et al. [29] proposed a ball mill-assisted solid acid hydrolysis of cellulose, yielding CNCs with good thermal stability and aspect ratio. The solid acid can be easily recovered by recrystallization during the reaction, and the entire process can be conducted at room temperature for effective recovery.

Ferric chloride hexahydrate is often used in the preparation of CNCs because of its strong acidity, and the hydrated ferric ion can easily penetrate the cellulose molecular chain and destroy the hydrogen bond between the molecular chains. By treating lignocellulosic fibers with molten ferric chloride hexahydrate, CNCs can be quickly obtained at low temperatures. Through simple concentration and crystallization techniques, ferric chloride can be recovered from the wastewater with a high recovery rate of 94.0–94.5 wt.%, thereby reducing production costs and minimizing the generation of acidic wastewater [30].

Compared to conventional acid hydrolysis, solid acid hydrolysis offers advantages such as mild reaction conditions, recyclability, low corrosion, and ease of surface functionalization. However, its limitations include low reaction efficiency and a limited selection of suitable acids.

Ionic liquids have gained attention for the production of CNCs due to their attributes, including high solubility, chemical stability, thermal stability, and nonflammability [31]. Similar to acid hydrolysis, ionic liquids facilitate the cleavage of glycosidic bonds in cellulose, releasing dispersed CNCs. However, the presence of sulfate groups in the ionic liquid can lead to the deposition of sulfur on the CNCs’ surface, which may decrease the thermal stability of the product. To enhance the thermal stability of the CNCs, researchers have explored various desulfurized ionic liquids. Studies have revealed that the thermal stability of the CNCs is not only related to sulfur but also associated with the alkyl chain of the ionic liquid. The longer the alkyl chain, the higher the thermal stability of the CNCs. The reason for this may be that as the alkyl chain lengthens, its hydrophobicity increases, leading to weaker intermolecular forces between the alkyl chain and the cellulose molecular chains, thereby retaining a greater amount of ionic liquid within the CNCs [32]. While ionic liquids exhibit good solubility, not all ionic liquids have high solubility, prompting the consideration of adding other media to the ionic solutions to enhance solubility. Haron et al. [33] extracted CNCs from microcrystalline cellulose (MCC) using a [Bmim][HSO_4_]/DMSO binary solvent system. The study indicated that compared to the use of pure ionic liquids, the addition of dimethyl sulfoxide as a co-solvent can increase the yield of CNCs with high thermal stability. The ionic liquids currently in use are associated with high production and recovery costs and are mostly toxic, underscoring the need for further research into the development of efficient, inexpensive, and safe methods for CNC production.

2,2,6,6-tetramethylpiperidine-1-oxyl (TEMPO) oxidation represents a promising method for the acid-free preparation of CNCs, where TEMPO selectively oxidizes cellulose moieties in an alkaline medium to initiate a sustainable reaction chain. Zhou et al. [34] employed TEMPO to oxidize softwood bleached kraft pulp (SBKP) to produce oxidized cellulose. The subsequent sonication of the oxidized cellulose for varying durations resulted in the production of CNCs with different sizes. Akira et al. [41] further extended the application of TEMPO oxidation to lignin fibers, yielding three different forms of nanocellulose, including cellulose nanonetworks, cellulose nanofibers, and cellulose nanocrystals. The key to the preparation of these different kinds of nanocellulose is to control the strength and timing of mechanical forces. This method has brought about the expansion of the application field, and the suitable nanocellulose can be obtained by selecting the appropriate processing conditions according to different application needs.

In addition to TEMPO oxidation, other methods have been employed to decompose cellulose oxidatively. For instance, the use of irradiation techniques for the pretreatment of lignin results in the generation of radicals with high oxidation activity, which can efficiently oxidize lignin and hemicellulose. The hydroxyl groups in lignin and hemicellulose are oxidized to carboxyl groups, which are more readily separated using organic solvents. Subsequent ultrasonication results in the production of CNCs [35].

Liu et al. [36] reported an efficient method for obtaining CNCs, where potassium periodate in an alkaline environment preferentially oxidizes the disordered regions of cellulose, preserving the ordered regions to form homogeneous CNCs. Wang et al. [37] described a process for preparing needle-like carboxylated CNCs. Lignocellulose was treated with sodium periodate to produce bialdehyde cellulose, followed by the oxidation of the aldehyde groups to carboxyl groups using the Fenton oxidation reaction. The final product, needle-like carboxycellulose nanocrystals, was obtained through high-pressure homogenization.

The chemical method for preparing CNCs requires the disposal of waste liquid after the reaction, which can be environmentally unfriendly. To address this, Kang et al. [38] employed a wet ball milling technique, ball milling cellulose with water and then separating the CNCs via centrifugation. This method eliminates the need for strong acids or organic solvents, and the resulting CNCs exhibit thermal stability similar to that of the original cellulose, as well as surface functional groups that mirror those of the pristine cellulose. Najwa et al. [39] conducted the ball milling of MCC after ultrasonication and controlled milling parameters such as time and concentration to obtain well-dispersed, high-thermal-stability, and high-crystallinity CNCs. While this method avoids the use of strong acids, it may introduce metal ions during the ball milling process. Yao et al. [42] prepared CNCs from MCC using colloidal milling, which involved sieving the raw material and then mechanically milling it, varying the centrifugal speed to obtain CNCs of different sizes.

Bioprocessing is a safe and eco-friendly production method, with enzymatic hydrolysis being a viable option for CNC preparation. It is now widely recognized that enzymatic hydrolysis results from the synergistic action of three cellulases, which selectively target the amorphous regions while preserving the crystalline zones, thereby yielding nanocellulose with specific morphological and structural attributes [40]. As a bioprocessing technique, enzymatic hydrolysis necessitates investigation into the factors influencing enzyme reactions to determine the optimal conditions. This method is favored due to its low reaction efficiency, which can be enhanced by the physical or chemical pretreatment of the raw material [43]. Zhang et al. [44] developed a highly efficient mechanical enzymatic hydrolysis method by incorporating liquid-assisted milling, demonstrating that an appropriate amount of liquid can increase enzyme catalytic activity and facilitate cellulose hydrolysis. G Banvillet et al. [45] used sodium hydroxide to pretreat bleached eucalyptus pulp before enzymatic hydrolysis, finding that this pretreatment effectively prepared cellulose nanofibers and improved enzyme accessibility. Tong et al. [21] hydrolyzed cellulose using a composite enzyme system consisting of cellulase and xylanase, with the ratio of these enzymes significantly affecting the hydrolysis rate and the morphology of the CNCs. Overall, enzymatic hydrolysis offers environmental protection, low energy consumption, and a wide range of raw materials, but it also comes with higher costs, longer processing times, and potentially lower yields.

## 3. Liquid Crystal Phase Self-Assembly of Cellulose Nanocrystals

**Figure 2:**
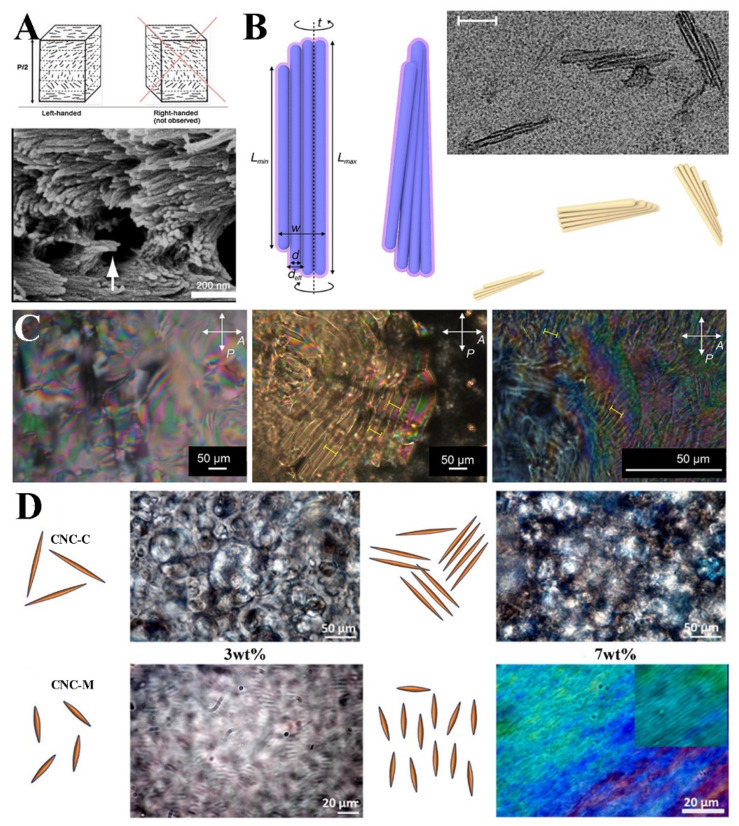
(**A**) Sketch of chiral nematic arrangement of CNCs and SEM image of CNCs’ film fracture surface [10]; (**B**) sketch of a generic chiral hard-splinter model and observations of CNCs. Scale bars correspond to a 100 nm length [18]; (**C**) polarized optical micrographs of isotropic liquid phase of 15 wt.% DSCG doped with 1 wt.% D-glucose, 1 wt.% pure CNCs, and 1 wt.% CNCs-DSCG mixture in water. Yellow marker lines indicate measured helical pitch locations [52]; (**D**) polarized optical microscopy (POM) images of CNCs-C and CNCs-M at different concentrations [19].

The preparation of CNCs illustrates that various hydrolytic methods selectively cleave the amorphous regions of cellulose, releasing the microcrystalline regions. CNCs represent the most distinctive structure within cellulose, characterized by a highly ordered arrangement. The structural formula of CNCs can be represented as C_6_H_10_O_5_, with a typical diameter ranging from 5 to 20 nm and a length of approximately 100 to 300 nm. The size and morphology of CNCs can vary depending on the raw materials and hydrolysis methods employed [46]. CNCs are expected to exhibit a liquid crystalline state due to their bar-like shape and size, and when the CNC concentration reaches approximately 3 wt%, phase separation occurs, resulting in the formation of an ordered cholesteric structure. In Figure 2A, the cholesteric structure and structure diagram of CNCs are shown [10].

Researchers have experimentally found that the liquid crystal behavior of CNCs is related to factors such as their shape, size, and surface charge density.

The liquid crystal behavior of CNCs in water was first observed by Marchessault et al. [47] in 1959. With the advancements in characterization techniques, the chiral liquid crystal properties of CNCs have been studied in greater depth.

Usov et al. [48] utilized a range of microscopy techniques and discovered that a broad spectrum of nanocelluloses display right-handed helical chirality. The torsion angle distribution of the cellulose was analyzed using statistical methods, revealing a non-Gaussian distribution, which suggests that the torsion may arise from the preparation process rather than from the transition between crystalline and amorphous regions. However, the mechanism underlying chiral transfer has not been fully elucidated.

To investigate the relationship between chirality and the shape of CNCs, Chiappini et al. [18] developed a model where CNCs were conceptualized as helical structures composed of multiple hard spherical prisms. Simulations were conducted to calculate the pitch of the liquid crystal phase of CNCs in a nonpolar solution, with the simulated data aligning with the experimental results, confirming that the shape of CNCs is intricately linked to their chirality. The model structure is shown in Figure 2B. The relationship between the shape and chirality of CNCs is expected to be further elucidated with the enhancement of observational techniques. Such insights may be achieved through innovative experimental approaches.

Based on cryo-transmission electron microscopy and electron microscopy diffraction techniques, the observation of CNCs reveals that their twisted geometric structure at the nanoscale is closely related to liquid crystal behavior. In aqueous solutions, CNCs maintain a continuous twist structure, exhibiting enhanced chiral optical activity. When the solution is dried, the continuous twist structure transforms into a sharp twist structure, at which point the chiral characteristics are weakened [49]. This is due to changes in the number and morphology of the CNCs’ kinks during the drying process, which in turn affects the chiral characteristics [50].

Goncalves et al. [51] functionalized the CNCs’ surface and discovered that the CNCs’ surface could effectively transmit chirality even after modification with achiral molecules, suggesting that the structural and morphological chirality of CNCs themselves are the primary sources of the transmitted chirality. 

Subsequently, Goncalves et al. [52] explored the CNCs’ ability to transfer chiral information to columnar phase liquid crystal systems. Comparing CNCs with molecular chiral molecules, it is found that CNCs exhibit higher chirality transfer efficiency. After the disodium cromoglycate (DSCG) modification of CNCs, it was observed that the rod-like structure of CNCs complements the DSCG stacking formed in the liquid crystal medium, which aids in chirality transfer. As can be seen from Figure 2C, the CNCs-DSCG-doped samples have a shorter helical pitch, indicating that they effectively enhance the interaction between liquid crystal molecules, thereby increasing the order and chirality of the liquid crystal. This further confirms the conclusion that the inherent structural and morphological chirality of CNCs are the primary sources for chirality transfer.

The specific impact of CNC length on self-assembly can be obtained through theoretical calculations. Raghuwanshi et al. [19] investigated the self-assembly behavior of CNCs of two distinct lengths in aqueous suspensions. They found that short CNCs transition from an isotropic phase to a nematic phase with increasing concentration, while long CNCs maintain an isotropic distribution. According to the calculations, this is due to the fact that long CNCs exhibit stronger van der Waals forces, which hinder self-assembly. However, this is merely a theoretical analysis of the self-assembly behavior of CNCs with different sizes, and it has not fully explored the impact of other property changes brought about by CNC size variations on self-assembly. Figure 2D is a microscope view of CNC self-assembled products of different lengths.

Surface charge plays a significant role in the stabilization of most suspensions and also has a substantial impact on the liquid crystal behavior of CNCs. Stroobants et al. [17] identified that charge plays a pivotal role in the liquid crystal phase transition of rod-like polyelectrolyte solutions. Intercharge repulsion increases the effective diameter and tends to align the rods vertically, producing a twisting effect. CNCs are materials composed of cellulose molecules, which exhibit a rod-like structure. Consequently, they can be regarded as rod-like polyelectrolytes. The specific electrostatic interactions involved in the self-assembly of CNCs require in-depth investigation.

Considering that the shape and length of CNCs are inextricably linked to their surface charge, Abitbol [53] examined the impact of surface charge density on the phase separation behavior and viscosity of CNC suspensions. It was determined that as the surface charge density increased, CNC suspensions were more likely to undergo phase separation at higher concentrations, forming tighter helical nematic phases, and the viscosity of the suspensions decreased. Therefore, the adjustment of the CNCs’ surface charge during self-assembly is a critical consideration. However, the dominant role of the CNCs’ shape and surface charge in the self-assembly process needs to be expanded.

**Figure 3:**
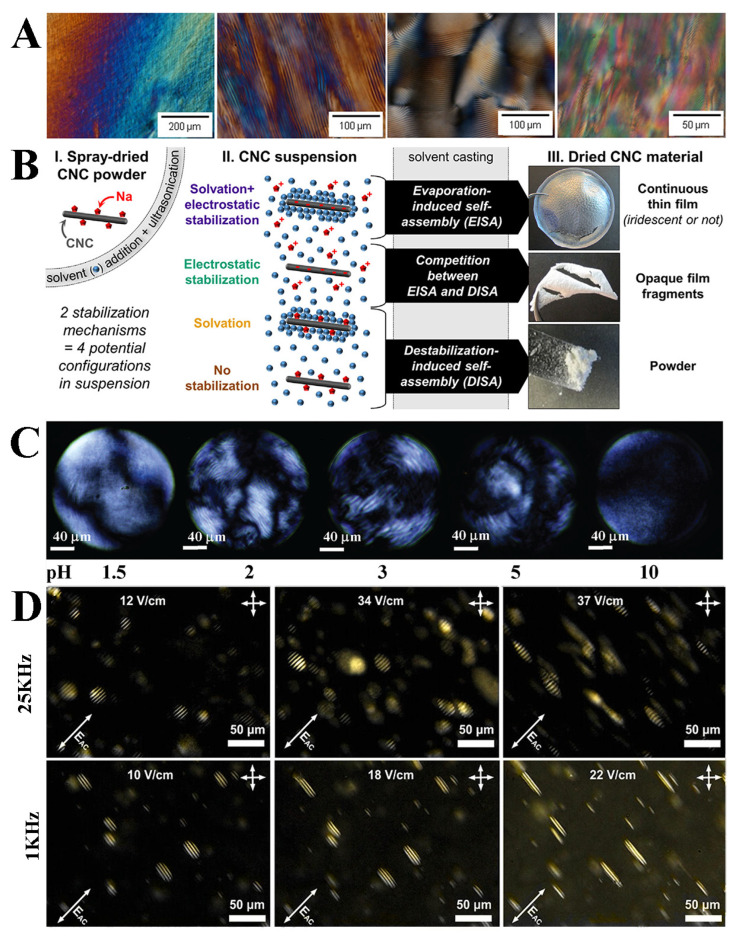
(**A**) Polarized optical images of cellulose nanocrystal suspensions in N, N-Dimethylformamide (DMF), water, formamide, and N-Methyl formamide (NMF) [54]; (**B**) self-assembly mechanisms of CNC suspensions stabilized by different mechanisms [55]; (**C**) POM images of CNC liquid crystals at pH = 1.5, 2, 3, 5, and 10 [56]; (**D**) polarized optical microscope photograph of CNCs’ spiral axis rotating parallel/perpendicular to the electric field as the electric field strength increases at high/low frequencies [58].

Bruckner et al. [54] explored the influence of diverse solvents on the self-assembly of CNC suspensions into helically oriented liquid crystal phases. They discovered that solvents with high dielectric constants significantly accelerated the self-assembly rate, reduced the concentration dependence of the helical period, and produced tighter helices. This is due to the solvent weakening the surface sulfate groups of the CNCs, resulting in an increase in the surface charge, which makes the CNCs more mobile and rearrangeable. In Figure 3A, the texture of the cellulose nanocrystal suspension with a mass fraction of 6% in DMF, water, formamide, and NMF is shown under a polarizer.

Bruel et al. [55] further delved into the impact of solvent parameters on the CNCs’ self-assembly. They determined that CNC suspensions can be categorized into distinct stabilization mechanisms based on the solvent dielectric constant and the CNCs’ chemical affinity. Under different solvents, evaporation-induced self-assembly (EISA) and destabilization-induced self-assembly (DISA) dominate the self-assembly process. The stabilization mechanism of CNC suspensions dictates their self-assembly behavior. Its principle is shown in Figure 3B. The influence of the solvent and surface charge on the self-assembly process of CNCs was further investigated.

Li et al. [56] examined the effect of varying pH on the liquid crystal phase transition of nanocellulose crystals. They revealed the presence of two nematic phases in low- and high-pH regions and an intermediate chiral nematic phase at intermediate pH. The microscope image is shown in Figure 3C, pH = 1.5, 2, 3, 5, and 10 from left to right. Adjusting the pH value will affect the ionic strength of the suspension. This finding corroborates the theory proposed by Stroobants [17] that electrostatic forces are pivotal in controlling the strength of the chiral nematic phase of CNCs.

After studying the intrinsic properties of CNCs, researchers have found that the influence of external forces on the self-assembly of CNCs cannot be neglected. Mao et al. [57] discovered that the pyranose ring on the CNCs’ molecular chain becomes magnetized under a magnetic field due to its permanent magnetization, leading to the CNCs’ molecular chain being subjected to magnetic forces. When the concentration of CNC suspensions reaches a phase separation threshold, the CNCs’ laminated structures of the nematic phase can be preferentially aligned along the magnetic field direction under a weak magnetic field, while the isotropic phase remains unaffected.

Qu and Zussman [58] demonstrated that the orientation and pitch of the helical axis of cholesteric CNCs can be modulated by adjusting the electric field strength and frequency. The CNC particles, under the influence of the electric field, generate an electric dipole moment that affects their movement. This electric field strength and frequency are highly applicable for tuning the CNC liquid crystal phase. As shown in Figure 3D, with the increase in the electric field intensity E at a low frequency, the CNCs’ spiral axis will become perpendicular to the electric field direction. At high frequencies, as the electric field intensity E increases, the CNCs’ spiral axis becomes parallel to the electric field direction. The control of the CNCs’ pitch by electric fields holds great significance for the future color regulation of CNC films.

Parton et al. [59] observed that chiral self-assembly in CNC suspensions is associated with specific subgroups of CNC particles. The CNC particles were categorized into four types based on their area-equivalent width and rectangularity: aggregates, bundles, crystallites, and distorted crystallites. Among them, the mechanism of action of the bundle subgroup in chiral doping is analogous to that of chiral dopants in molecular liquid crystals. It is a necessary condition for the formation of the helical phase in cellulose nanocrystal suspensions and can effectively control the pitch of the helical phase.

In summary, the exact origin of the chiral self-assembly of CNCs remains controversial. The shape, charge density, and other aspects of CNCs have a significant combined effect on their self-assembly, and further research in this area is needed to elucidate the precise source of the chiral self-assembly of CNCs.

## 4. Cellulose Nanocrystal Photonic Crystal Film

As the study of the liquid crystal phase of CNCs has progressed, it has been observed that when the pitch of the helix distortion aligns with the wavelength of light, the interference of the light can be observed. This interference phenomenon of the incident light is a key factor in the formation of structural colors in CNCs.

In 1992, Revol et al. [60] discovered that CNCs spontaneously separate into two phases in suspension at a critical concentration: an ordered anisotropic phase and a disordered isotropic phase. Upon solvent evaporation, CNCs solidify into a film, maintaining their helical structure. This chiral film exhibits distinct optical properties [61]. According to the Bragg diffraction equation, the reflection diffraction of CNC photonic crystal films is related to the spacing of the CNCs’ chiral structure [16]. Consequently, adjusting the spacing of the CNCs’ chiral structures can yield flexible structured color films.

### 4.1. Preparation of Cellulose Nanocrystalline Photonic Crystal Films

The preparation of CNC photonic crystal films commences with the CNCs’ dispersion, where the CNCs are uniformly dispersed in an isotropic solution. As the solvent evaporates, the CNCs’ dispersion undergoes phase separation at a CNC concentration of 3% by weight, spontaneously forming two distinct phases: isotropic and anisotropic.

**Figure 4:**
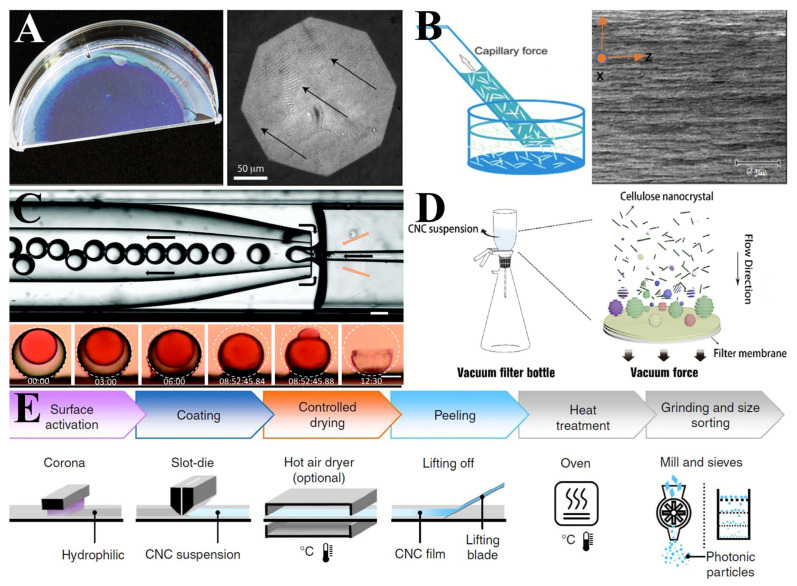
(**A**) A digital photograph of the CNC film and optical micrographs of tactoids. The arrows highlight the positions of the tactoids. [62]; (**B**) schematic diagram of the capillary-induced self-assembly method and SEM cross-section analysis of capillary-formed CNC films [66]; (**C**) Microscopic photo of CNC shell production using microfluidic device (upper part) and side views of a CNC shell with dyed inner phase during drying (lower part) [69]; (**D**) schematic diagram of the vacuum-assisted self-assembly method [71]; (**E**) flowchart of the key steps in the preparation of photonic crystals by the R2R method [75].

The evaporative self-assembly process of the CNC suspension is generally considered to be divided into three stages. In the initial phase, CNCs are uniformly suspended, and the interface between the air and the CNC suspension is directly imaged. At this stage, the reflected light spectrum is homogeneous with a reflectance of 2%. In the second phase, as the solvent evaporates, the liquid becomes viscous, and the interface transitions from the gel–air to the gel–culture dish, with enhanced reflectivity. The stacking structure of CNCs is also discernible. The optical photograph of the CNC film and the microscope image of viscous tactoids are shown in Figure 4A. In the final phase, when the water is completely evaporated, the CNCs’ stacking structure is immobilized as a helical arrangement of liquid crystals [62].

Therefore, extending the evaporation time after the CNC suspension has reached the liquid crystal state can enhance the uniformity of the resulting thin film. During the phase separation and gelation stages, an extended evaporation time allows the stacked structures of CNCs to rearrange and fuse, resulting in highly ordered and homogeneous CNC films. Presently, the self-assembly of CNCs necessitates sufficient time, thus necessitating prolonged evaporation to maintain stability [63].

Achieving high-quality, uniform CNC films have consistently been a focal point of research. At present, aside from the long time investment required for the film formation process, the coffee-ring effect during evaporation poses an urgent challenge to be solved. This effect is the accumulation of nanoparticles at the three-phase contact line, resulting in the formation of a concentration gradient at the edge. This concentration gradient results in a thicker film in the edge region and a thinner film in the center region, forming a ring-like pattern. The coffee-ring effect can be overcome by introducing shear flow in the film drying process or adding substances with a strong water absorption ability to enhance the hygroscopic property of the film [64].

For non-circular regions, the coffee-ring effect may facilitate some specialized applications. Studies indicate that in non-circular CNC films, the coffee-ring width at the corners is greater than that at the edges. The larger the film area, the smaller the discrepancy in the coffee-ring width between the corners and the edges. Utilizing these characteristics allows for the acquisition of iridescent thin-film fragments with specific shapes [65].

Selecting specific drying regions has opened up a new avenue of research for the preparation of uniform CNC films. Utilizing the capillary drying of CNC suspensions enables the rapid fabrication of uniform chiral CNC solid thin films. Figure 4B shows the process of CNC capillary-induced self-assembly and shows SEM cross-section analysis of capillary-induced self-assembly. In comparison to conventional evaporation-induced self-assembly, the samples prepared by capillary-drying self-assembly exhibit a higher orientation, birefringence, and reflective bandwidth [66].

In addition to cylindrical capillaries, within square capillaries, a concentrically arranged lamellar structure is formed, exhibiting square layers adjacent to the tube walls, while tending towards cylindrical layers in the central region. In smaller capillaries, CNCs do not conform to square bending and form concentric cylindrical layers. As the inner diameter of the capillary increases, the concentric arrangement of the CNC layers assumes a spiral shape [67].

By expanding the confined area, CNCs self-assemble to produce microscopic lamellar structures. The self-assembly process can be divided into two stages: a rapid drying phase at the edges and a slow diffusion phase in the center. Due to the high concentration of CNCs at the edge regions and the corresponding fast evaporation rate, lamellar structures are formed first. As the lamellae at the edge regions are established, the concentration of CNCs in the central area gradually decreases, leading to a slower evaporation rate, with diffusion becoming the predominant mass transfer mechanism. The self-assembly process ultimately results in the formation of an ordered lamellar structure across the entire droplet. As the solvent completely evaporates, the lamellar structure is solidified [68].

Utilizing microfluidic techniques, the acquisition of spherical structures is feasible, offering a promising solution to the issues of wrinkling and deformation that arise from direct drying. By selecting CNCs with a high aspect ratio as the middle phase and employing an inert oil as the inner and outer phases, the formation and stabilization of the liquid crystal phase are facilitated, thereby enhancing the optical properties of the shell. The immiscibility of the inert oil with the CNC suspension does not interfere with the liquid crystal behavior of the CNCs, thereby ensuring the stability and uniformity of the liquid crystal phase within the shell. A co-flow microfluidic device is utilized to prepare double-emulsion shell structures. Following drying, color-tunable CNC spheres can be obtained [69]. Figure 4C illustrates the procedure and drying process for the fabrication of spherical CNCs using the microfluidic method.

The preparation of CNC films within confined regions satisfies the demand for high-quality products; however, the fabrication of large-area, high-quality films remain a necessity. The layer-by-layer Langmuir–Schaefer assembly technique can be employed to produce large-area, highly ordered thin films. This method allows for precise control over the thickness and number of layers of the film, and it offers a broad range of materials for selection. The nanomaterials to be fabricated are dispersed on the water surface, and by compressing the barriers in the Langmuir trough, the material is formed into a monolayer at the air–water interface. A pre-prepared substrate material is placed beneath the Langmuir trough, and by controlling the movement of the barriers, the monolayer at the air–water interface is transferred onto the substrate, thereby forming the film [70].

The acquisition of large-area, high-quality, and low-cost CNC films is efficiently achieved through the use of vacuum-assisted self-assembly (VASA). This technique exploits the strong pressure differential in a vacuum environment to rapidly expel the solvent from the solution, leaving the solid particles on the surface of the filtration membrane, thereby facilitating the rapid assembly of the material. Compared to the traditional evaporation-induced self-assembly (EISA) method, which typically requires several days for material preparation, VASA technology can complete the process in just a few hours. Moreover, the films produced by VASA exhibit fewer defects, smoother surfaces, and more uniform structural colors when compared to those produced by the EISA method. A schematic illustration of this process is depicted in Figure 4D [71].

During VASA, research has found that the chiral axis of CNCs aligns with the hydrostatic pressure direction under vacuum force. Through filtration, the concentration of CNCs increases, leading to the formation of more small nematic phase structures that eventually fuse into long-range periodic layers with left-handed chiral nematic structures [72]. Similar to the principle of VASA, pressure-assisted self-assembly (PASA) also enables the fabrication of high-quality CNC films. The CNC suspension is placed in a vacuum filtration cup and subjected to pressure application. The vertical pressure induces the deposition of CNCs and the formation of an orderly arrangement, thereby constructing a chirality liquid crystalline phase with a regular structure. Compared to VASA, PASA offers a broader range of applicability and is simpler to operate [73].

For EISA, considerable advancements have also been made by optimizing solvents, evaporation processes, and the substrate. The manipulation of the substrate during the evaporation process through rotation yields CNC films with unique structures. In static evaporation, CNC molecules tend to orient parallel within certain nematic phases. In dynamic rotation, the centrifugal force generated by the rotation induces the CNC molecules to orient towards the center of rotation. The structure and optical properties of the CNC films can be modulated by controlling the rotation speed and direction [74].

Drogue et al. [75] investigated a method for the large-scale preparation of CNC photonic crystal films. Large-sized color structured films were fabricated using a roll-to-roll coating process. The process flow is shown in Figure 4E. Polyethylene terephthalate (PET) substrates were treated with plasma etching or corona discharge to enhance their surface energy and facilitate the wetting of CNC suspensions. The ultrasonicated CNC suspension was then applied to the treated PET substrate and dried using a hot air blower to produce large-scale CNC photonic crystal films. It is essential to control the evaporation rate of the solvent during the preparation process, as a slower drying rate promotes CNC particle self-assembly, while a faster drying rate inhibits self-assembly and degrades the color of the CNC films.

**Figure 5:**
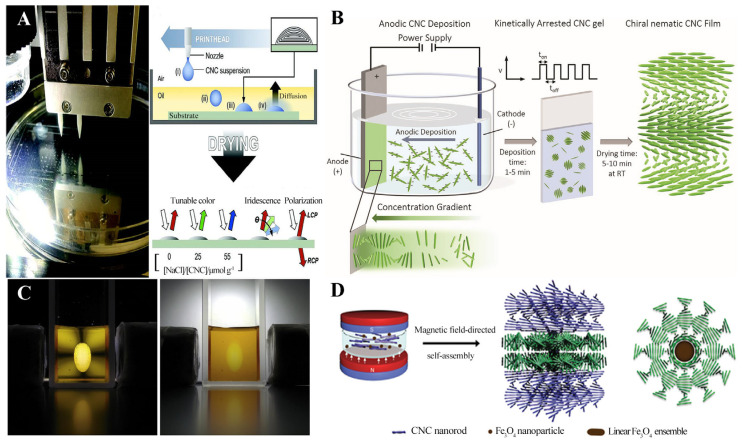
(**A**) A photo of the inkjet printing device (**left**) and a diagram of the printing process (**right**) [76]; (**B**) schematic of the electrophoretic deposition, set-up, and the self-assembly process of the electrodeposited chiral nematic CNC film [79]; (**C**) the left image shows that the concentration of magnetic nanoparticles in the isotropic phase is higher than that in the tactoids due to repulsion. The right image illustrates the four forces acting on the tactoids in a magnetic field [81]. (**D**) The process of utilizing the self-assembly of Fe_3_O_4_ nanoparticles under an external magnetic field to obtain CNCs-Fe_3_O_4_ composite films with planar–nematic dual texture and circularly polarized optical properties [82].

Inkjet printing, as the most commercial production route, has also demonstrated advantages in the preparation of CNC films. By adding various additives to the CNC suspension, different printing inks are obtained. These additives effectively modulate the color of the CNC films. To circumvent the coffee-ring effect, a layer of oil is overlaid on the CNC droplets, transforming the mode of water loss from evaporation to diffusion, as shown in Figure 5A. Due to the low saturation concentration of oil for water, the rate of water loss is significantly reduced, thereby extending the drying time and suppressing the coffee-stain effect, ultimately resulting in the formation of microfilms with uniform coloration [76].

In addition to conventional methods, various pathways to facilitate the acceleration of CNC self-assembly have been extensively investigated. Utilizing alternating current to assist CNC self-assembly is recognized as an efficient method.

Research has achieved a highly uniform orientation and alignment of cellulose nanocrystal suspensions by applying an alternating voltage between parallel metal electrodes. Under the influence of the electric field, CNCs generate an induced dipole moment, and the electric field force acts on this induced dipole, causing the CNCs to align along the direction of the electric field. 

Kasuga et al. [77] prepared CNCs with varying orientations at the anode by modulating the voltage between electrodes using electrophoresis and electrochemical deposition. Atifi et al. [78] employed an electrophoretic deposition technique to rapidly and efficiently produce homogeneous CNC photonic crystal films. Due to the negative charge of CNCs, they migrate towards the positive pole during the deposition process, depositing into a gel layer. This gel layer is then dried to yield a solid film, a process that typically takes 10 min. The preparation diagram is shown in Figure 5B. The method of fabricating CNC films using an alternating electric field effectively suppresses the coffee-ring effect. The alternating electric field induces periodic oscillations of the contact angle at the droplet surface, leading to periodic changes in the droplet shape and thereby inhibiting the formation of the coffee ring. There exists an optimal frequency at which the dynamics of the three-phase line reach an equilibrium, resulting in the formation of a uniform ultrathin film. For CNC films, this optimal frequency is approximately 1 kHz [79].

Accelerating the self-assembly of CNCs using a magnetic field is a convenient approach. Magnetic fields can effectively enhance the assembly process of CNCs, and Granston and Gray [80] found that CNCs exhibit negative anisotropic magnetization, indicating that in a magnetic field, the nanocrystals align parallel to the direction of the magnetic field. When CNC suspensions are subjected to a magnetic field, the CNCs align along the direction of the magnetic field. Experiments have shown that the addition of a magnetic field significantly reduces the orientation alignment time of nanocrystals, thereby improving the efficiency of self-assembly.

Wang et al. [81] modified the magnetization rates of the isotropic and anisotropic phases by incorporating magnetic particles into the CNC suspension. Under the influence of a gradient magnetic field, the disordered phase moves towards the high-magnetic-field region, while the liquid crystal microphase moves towards the low-magnetic-field region. A quadrupolar magnetic field is employed to create a magnetic trap that confines the liquid crystal microphase at a specific location for accelerated self-assembly. Figure 5C illustrates how magnetic buoyancy affects the movement and orientation of liquid crystal tactoids in a gradient magnetic field. The left image depicts the repulsive interaction of liquid crystal tactoids with superparamagnetic doped nanoparticles, resulting in a higher concentration of superparamagnetic doped nanoparticles in the isotropic phase than in the tactoids. Consequently, the magnetic susceptibility of the isotropic phase is significantly higher than that of the tactoids. The right image illustrates the four forces acting on the tactoids in a magnetic field, where the competition between gravity and magnetic force influences the position of the tactoids’ movement.

Under the magnetic field, the participating Fe_3_O_4_ particles form nano-chains along magnetic susceptibility lines, generating localized magnetic fields. These localized magnetic fields induce the CNCs surrounding them to form helical axis orientations of isotropic circles around the Fe_3_O_4_ linear assemblies. Li et al. [82] reported the use of localized magnetic-field-guided self-assembly of CNCs to prepare cellulose nanocrystalline thin films with left-handed helical nematic columnar phases. This magnetic-field-induced Fe_3_O_4_ self-assembly was employed to achieve effective control of the CNCs’ helix axis orientation from within the CNCs. Figure 5D summarizes the process of using the self-assembly of Fe_3_O_4_ nanoparticles under an external magnetic field to control the nematic phase orientation and pitch distribution in CNC films so as to obtain a CNCs-Fe_3_O_4_ composite film with a planar–nematic double texture and circular polarization optical properties.

The current research on the preparation of CNC photonic crystal films necessitates rapid, efficient, large-area, and high-quality fabrication. Conventional evaporation-induced self-assembly is limited by its long processing time and significant concentration gradients within the suspension, leading to the poor uniformity of the photonic crystal films. To enhance the self-assembly speed and film quality, current strategies include improving the surface properties of substrates and introducing external force fields.

### 4.2. Structural Color of Cellulose Nanocrystalline Photonic Crystal Films

From the previous section, it can be observed that the drying film formation process of CNCs can be categorized into three distinct stages. The formation of the helical stacking of CNCs occurs when the CNC suspension transitions to a viscous liquid state following the evaporation of the solvent. According to the Bragg diffraction equation, the helical pitch is recognized as the primary factor governing the color change in the film. In the viscous liquid phase, parameters such as the CNC concentration, evaporation rate, CNC size distribution, surface charge density, and ionic strength can significantly influence the CNCs’ pitch.

According to phase separation studies of CNC suspensions [83], the pitch typically decreases with increasing particle concentration. A higher CNC concentration results in stronger interaction forces between the CNCs during self-assembly, leading to a reduction in the layer spacing of the helical lamellar structure, i.e., a decrease in the pitch. During the CNC film formation process, reducing the evaporation rate can regulate the CNC concentration in the viscous phase, thereby enabling the control of the pitch and resulting in a redshift of the CNC film. Two methods are commonly employed to control the evaporation rate: traditional temperature control, which prolongs the viscous droplet stage by decreasing the evaporation temperature, and the addition of other substances to induce the CNC suspension to reach a gel-forming concentration.

**Figure 6:**
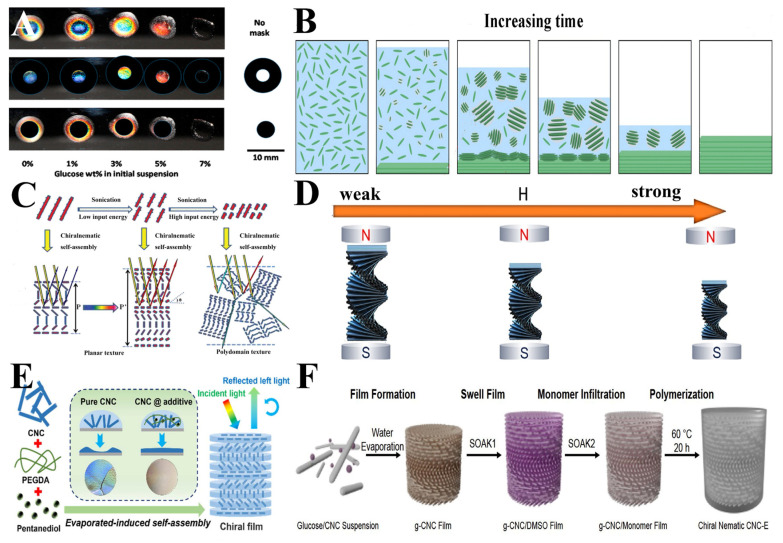
(**A**) Structural color of CNC suspensions with glucose added from 0 to 7 *w*/*v* % concentration. The following two images are masked to show the color of the center and edges of the dried droplets [84]. (**B**) Schematic illustration of a CNC suspension forming a CNC film as water evaporates [86]; (**C**) cholesteric phase transformation of cellulose nanocrystalline with different sizes [88]; (**D**) screw pitch variation in CNCs/Fe_3_O_4_ nanoparticles in magnetic fields of different intensities [91]; (**E**) schematic diagram of preparation of ternary supramolecular (CNC/PEGDA/pentanediol) by evaporation-induced self-assembly [95]; (**F**) schematic representation of the preparation of chiral nematic cellulose nanocrystal elastomers [97].

By adding substances similar in structure to CNCs, it ensures that there is no interference with the self-assembly of CNCs in the first stage. However, during the second stage of evaporation, it exhibits a plasticizing effect, reducing the CNC suspension to the concentration required for gel formation, thereby extending the evaporation time in the second stage. Ultimately, this results in a larger pitch for the modified CNCs compared to the unmodified pitch [84]. Figure 6A shows the reflected color of different concentrations of glucose after drying in CNC suspension. The following two images are obscured to show the color of the center and edge of the dried droplets. The color moves toward the red end as the glucose concentration increases. The selective removal of solvents is also a choice for controlling the evaporation rate. By dispersing CNCs in a mixed solvent and undergoing rapid evaporation, a nematic phase of CNCs with distinct upper and lower layers can be obtained after the evaporation of one type of solvent [85].

An electrolyte was also introduced into the CNC suspension to achieve the shielding of the CNCs’ surface charge and to control the pitch of the suspension. The study found that divalent cations, with their higher valence state, can more effectively interfere with the electrostatic interaction between CNCs and the positive ions in the solution, leading to the aggregation of CNCs and a reduction in pitch. Moreover, larger anions can more effectively compress electrostatic repulsion and reduce the pitch compared to smaller anions [86]. A schematic diagram of the CNC suspension forming a CNC film when water evaporates is shown in Figure 6B. In addition to inorganic salts, non-adsorbing electrolytes can also control the self-assembly of CNCs by influencing the surface charge of CNCs [87].

The size of CNCs also has a significant impact on the pitch. According to research, the smaller the average size of the CNCs, the greater the helical pitch of the film and the redshift of the reflected wavelength. Researchers have obtained regular data on the relationship between size and optical properties by using ultrasonic treatment to adjust the size of CNCs, as shown in Figure 6C. However, the CNC suspension treated with ultrasound cannot avoid changes in the viscosity, ionic strength, and surface charge of CNCs, making it impossible to confirm the dominant factor controlling the pitch [88,89].

There is an obvious relationship between the surface charge and pitch. When the surface charge density of the CNC suspension is high, the electrostatic repulsion is strong, and the critical concentration for the self-assembly of CNCs into helical layer structures is higher, resulting in a smaller pitch. An increase in the critical concentration leads to an increase in the number of CNC nanorods in each layer of the helical layer structure formed by self-assembly, while the total number of layers decreases, thereby increasing the pitch. In summary, the surface charge affects the critical concentration for the self-assembly of CNCs into helical layer structures, which in turn affects the pitch and the reflected wavelength, ultimately leading to changes in structural color [90].

Chen et al. [91] synthesized Fe_3_O_4_ nanoparticles in situ on the CNCs’ surface to produce Fe_3_O_4_/CNC composites. These composites were mixed with CNC suspension and dried into a film under a weak magnetic field. The pitch is controlled by compressing the arrangement of CNCs through the application of a magnetic field. Figure 6D shows the simulation diagram of the change in the pitch of the CNCs as the magnetic field increases. The superparamagnetic nature of the Fe_3_O_4_/CNC composites allowed the magnetic dipole force between the Fe_3_O_4_ nanoparticles to align the CNCs more closely, thereby reducing the pitch.

In addition to modulating the pitch of CNC self-assembly by altering the relevant properties of CNCs, other substances can be added to achieve pitch regulation. For example, in the CNC suspension, substances that can fill voids are doped. These doped substances are uniformly dispersed in the chiral liquid crystal of the CNCs, thereby changing the pitch to control the structural color. At the same time, these substances will endow the composite material with additional properties. However, this method requires research on the concentration of the added substances to avoid factors such as concentration that may destroy the structure of the CNCs [92].

Other natural macromolecular materials, such as water-soluble chitosan, which has a rich hydroxyl and amino group structure, can interact with CNCs to affect the distance and arrangement between CNC particles, thereby regulating the pitch and reflection wavelength of the CNC liquid crystal structure [93].

Lamellar assembly is a new strategy for preparing flexible photonic crystal films, which involves depositing materials layer by layer to construct a multilayer structure. By selecting substances that improve the rheological properties of CNCs, a multilayer film can be obtained by alternately depositing CNCs and this substance on the substrate. This film can be precisely controlled in terms of the pitch, color, and mechanical properties and is easy to prepare [94]. This method can also improve the strength of the CNC film and achieve multiple responsiveness.

Responsive materials are chemically bonded to the CNCs and are uniformly distributed in the liquid crystal phase of the CNCs. When stimulated by conditions such as water, alcohols, etc., these responsive materials undergo phenomena of water absorption or shrinking. In this case, the pitch of the entire CNC film will also stretch or shrink along with the changes in the responsive materials.

Common flexible polymer additives such as PEGDA, PEG, PVA, etc., are used to improve the flexibility of the film while the CNCs act as chiral templates to provide the foundation for photonic crystals. These flexible polymers participate in chemical bond networks to achieve the structural stability of the film. Jia et al. mixed PEGDA, CNCs, and glycol to prepare a ternary supramolecular ensemble. The preparation process is shown in Figure 6E. This film has a good response to solvents and humidity [95].

Dong et al. [96] introduced a novel self-assembly strategy to manipulate the photonic structure of the film by incorporating cationic polymers, poly (dimethyldiallylammonium chloride) (PDDA), and glycerol, leading to a color change. PDDA disrupted the long-range ordered structure of the CNCs, resulting in a non-angle-dependent structural color. Glycerol was inserted into the CNC array through hydrogen bonding, altering the pitch. An increase in the glycerol content further increased the helical period and accentuated the redshift effect. The precise control of the structural color of CNCs could be achieved by adjusting the glycerol proportion.

Boott et al. [97] presented an alternative approach by blending CNCs with D-glucose and subsequently evaporating to obtain a CNC membrane. The CNC membrane was then immersed in a zodiisobutyronitrile/Dimethyl sulfoxide solution. The preparation method is shown in Figure 6F. The addition of a solution containing ethyl acrylate and 2-hydroxyethyl acrylate monomers enabled full absorption. Polymerization was initiated at 60 °C using AIBN as a free radical initiator, resulting in CNC elastomers that maintained the helical structure. Under mechanical stress, the chiral nematic structure of the CNCs in the elastomers retained a degree of order while compressing the helical pitch. When the helical pitch fell within the visible range, the light of the corresponding wavelength was selectively reflected, causing a color change in the CNC elastomer.

The manipulation of the color in CNC photonic crystal films fundamentally relies on the adjustment of the helical pitch of the CNCs, in addition to the incorporation of other pigmented materials within the suspension. The addition of polymers to CNC suspensions not only facilitates control over the helical pitch but also enhances the strength and toughness of the resulting CNC films. However, the fabrication of CNC-based composite films introduces a new challenge, namely, large-scale and efficient self-assembly. To date, CNC-based composite films are predominantly produced on a small scale. Consequently, refining the self-assembly methodology for CNC composite films to meet the requirements of large-area, high-quality production is a focal area for future research.

## 5. Summary and Outlook

In this review, we review the preparation of CNCs, the self-assembly of CNC suspensions, and the film formation of CNC rainbow films. We have demonstrated that CNCs are an excellent type of novel liquid crystal material that not only possesses superior optical properties but also has the ability to self-assemble into photonic crystal structures. Currently, the preparation of CNCs involves chemical, physical, and biological methods, all of which involve the destruction of the amorphous region of cellulose to obtain nanocrystalline cellulose. Among them, the chemical method facilitates surface functionalization but results in significant environmental and equipment pollution. Biological and physical methods are more environmentally friendly, but they are disadvantaged in terms of cost and product quality. To obtain CNCs with low cost and low pollution, researchers are considering blending these three methods to develop a more advantageous preparation method.

The self-assembly mechanism of CNC suspensions during the drying process is a subject of significant interest. CNC suspensions can spontaneously form a left-handed chiral nematic phase at specific concentrations. After drying, the spontaneously formed left-handed chiral nematic phase becomes fixed within the CNC film, resulting in iridescent colors. These properties make CNCs suitable for applications in various fields such as coatings and inks, food packaging, composites, and more. Numerous perspectives exist on the mechanism by which CNC suspensions self-assemble into helical structures at high concentrations. For instance, the chiral shape of CNC particles themselves can facilitate the transfer of chirality. Factors such as the surface charge of the CNCs, the aspect ratio of the particles, the ionic strength, and the dielectric constant of the solution all exhibit an influence on the self-assembly of CNC suspensions. The roles of these factors in the self-assembly process require further in-depth investigation. Research on the self-assembly of CNCs can lay the foundation for the subsequent production of CNC films with structural color. In recent years, research on CNC materials has achieved many accomplishments in various fields, especially in optical applications. Ensuring the self-assembly of CNCs while obtaining films with a uniform morphology is an essential technique for practical applications. Methods ranging from traditional evaporation-induced assembly to capillary evaporation-induced assembly, and from vacuum-assisted to electrophoretic deposition, have been developed to achieve uniform iridescent CNC films. However, it is still in its infancy, and many issues remain to be addressed. Currently, the evaporation-induced strategy for film formation takes a long time, and more efficient film formation methods need to be developed to achieve fast film formation, uniform color, and precise control. The vacuum-assisted electrophoretic deposition method has reduced the film formation time, but it faces issues such as high production costs and a small film formation area. Currently, the priority is to combine these methods and extend them to commercial production.

CNCs possess strong mechanical properties, but the strength of CNC films is far from ideal. Researchers have prepared composite films by adding other functional materials to the CNC suspension. This method can control the pitch of the CNC film and regulate the color of the film, and it can also effectively improve the mechanical properties of the CNC film, which is a very promising approach. However, with the introduction of new materials, ensuring the uniform self-assembly of the CNCs has once again become a challenge.

CNCs, as environmentally friendly materials, have great potential for development. We look forward to future research based on the self-assembly of CNCs providing more inspiration and creativity for the application of CNCs. Researchers in this field will further advance the development of CNCs in self-assembly and photonic crystals.
